# The effect of adolescent inhalant abuse on energy balance and growth

**DOI:** 10.1002/prp2.498

**Published:** 2019-07-30

**Authors:** Rose Crossin, Ashleigh Qama, Zane B. Andrews, Andrew J. Lawrence, Jhodie R. Duncan

**Affiliations:** ^1^ Florey Institute of Neuroscience and Mental Health Parkville VIC; ^2^ Turning Point, Eastern Health Richmond VIC; ^3^ Eastern Health Clinical School Monash University Box Hill VIC; ^4^ WHO Collaborating Centre for Viral Hepatitis Peter Doherty Institute for Infection and Immunity Melbourne VIC; ^5^ Monash Biomedicine Discovery Institute Monash University Clayton VIC; ^6^ Florey Department of Neuroscience and Mental Health University of Melbourne Parkville VIC

**Keywords:** addiction, adolescence, endocrine, energy balance, glycemic control, growth, inhalant abuse, metabolism, substance abuse, toluene, volatile solvent abuse

## Abstract

The abuse of volatile solvents such as toluene is a significant public health concern, predominantly affecting adolescents. To date, inhalant abuse research has primarily focused on the central nervous system; however, inhalants also exert effects on other organ systems and processes, including metabolic function and energy balance. Adolescent inhalant abuse is characterized by a negative energy balance phenotype, with the peak period of abuse overlapping with the adolescent growth spurt. There are multiple components within the central and peripheral regulation of energy balance that may be affected by adolescent inhalant abuse, such as impaired metabolic signaling, decreased food intake, altered dietary preferences, disrupted glucose tolerance and insulin release, reduced adiposity and skeletal density, and adrenal hypertrophy. These effects may persist into abstinence and adulthood, and the long‐term consequences of inhalant‐induced metabolic dysfunction are currently unknown. The signs and symptoms resulting from chronic adolescent inhalant abuse may result in a propensity for the development of adult‐onset metabolic disorders such as type 2 diabetes, however, further research investigating the long‐term effects of inhalant abuse upon energy balance and metabolism are needed. This review addresses several aspects of the short‐ and long‐term effects of inhalant abuse relating to energy and metabolic processes, including energy balance, intake and expenditure; dietary preferences and glycemic control; and the dysfunction of metabolic homeostasis through altered adipose tissue, bone, and hypothalamic‐pituitary‐adrenal axis function.

**Abbreviations**
ACTH: adrenocorticotropic hormone
CNS: central nervous system
CRH: corticotropic-releasing hormone
GABA: gamma‐Aminobutyric acid
GTT: glucose tolerance test
HPA: hypothalamic-pituitary-adrenal
NMDA: *N*‐methyl‐D‐aspartate
NPY: neuropeptide Y
PYY: peptide YY
ppm: parts per million

## 1 AN OVERVIEW OF INHALANT ABUSE

Inhalant abuse is a form of substance abuse and involves the intentional inhalation of vapors from household or industrial products, in order to create a feeling of euphoria and an altered mental state.^1^ Products that are commonly abused as inhalants include petrol, spray paint, thinners, aerosols, and glue. These products are cheap, legal to purchase, and readily accessible, which is thought to add to their attractiveness as substances of abuse.^2^ While inhaled products are not technically considered “drugs,” they share the reinforcing nature of many drugs of abuse due to the presence of toluene, which is common to all these products. Petrol is approximately 7% toluene, whereas some spray paint and thinners can be up to 35% toluene, with highly variable percentages in glues and adhesives,^3^ though inhalant users report preferring pure toluene when it is available.^4^ Toluene is a volatile solvent; a colorless, water‐insoluble aromatic hydrocarbon, and is considered to underlie the addictive drive to continue abusing inhalants, therefore, toluene is commonly used as an experimental preclinical model for inhalant abuse. Thus, in this review, “inhalants” and “toluene” are used interchangeably, depending on whether reference is being made to clinical or preclinical studies.

Inhalant abuse is commonly known as “chroming”, “huffing”, or “bagging” as typical self‐administration involves inhaling concentrated vapors from spray cans, off solvent‐soaked cloth, or directly from a bag, respectively. When administered in these ways, the concentration of inhaled toluene is estimated to range from 3000 to 15 000 parts per million (ppm) (equivalent to 0.03‐0.16 mol/L).^5^ While the abuse of inhalants generally occurs for a short duration, ranging from minutes to 1 hour, for the majority of individuals this behavior is repeated over time and extends for periods greater than 1 year, such that the exposure becomes chronic but intermittent in nature.^6^ It is also important to note that, as well as intentional inhalation, exposure to inhaled toluene can also occur in industrial settings (eg, in factories where toluene is used as a solvent). Both the patterns of use and concentrations of inhaled toluene differentiate inhalant abuse from occupational exposure, with occupational exposure characterized by sustained contact to concentrations of toluene less than 200 ppm.^7^, ^8^ While this review focuses on inhalant abuse, it is acknowledged that some of the data pertaining to the toxicology of toluene has been determined using occupational exposure studies and these should be interpreted with caution relative to effects in the abuse setting; these studies are highlighted when discussed.

## 2 ADOLESCENT INHALANT ABUSE

**Figure 1:**
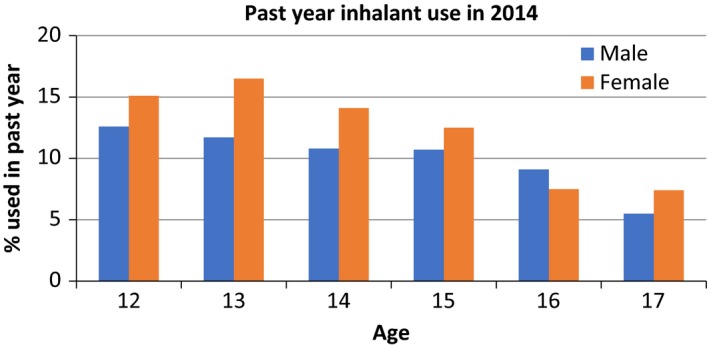
Past year inhalant use in Australian secondary school students, by gender, based on data sourced from the 2014 Australian Secondary School Drug Survey. The data highlight that, amongst all adolescents, the predominant age group misusing inhalants is young adolescents, with use decreasing as age increases. Figure adapted from data provided in^9^

Inhalant abuse is predominantly an issue in young adolescent populations (Figure 1), with prevalence rates approximately equal between males and females.^9^ Inhalants are one of the first and most common substances abused by the 12‐13 age group in Australia, with 14% of 12‐year‐olds having misused inhalants in the previous year, and of those, approximately 20% have misused inhalants 10 or more times in that year.^9^, ^10^ This pattern of early adolescent use is consistent globally.^11^ A recent survey by the Australian government found that, overall, inhalant abuse in Australia is increasing, with rates of recent inhalant use doubling more between 2007 and 2016 compared to a 4% reduction in recent alcohol and cigarette use over the same period.^10^ Similarly, the most recent United States Monitoring the Future Survey also reported a significant increase in recent use in Grade 8 students (aged 13‐14) along with a decline in the perceived risk of inhalant misuse.^12^

## 3 THE EFFECTS OF INHALANT ABUSE ON ENERGY BALANCE

The effects of inhalants on the central nervous system (CNS), including cognition, have been relatively well studied,^13^, ^14^, ^15^ and it is thought that inhibited *N*‐methyl‐D‐aspartate (NMDA) receptor signaling and region‐specific changes to glutamatergic and gamma‐Aminobutyric acid (GABA)‐ergic signaling underpin these effects.^16^, ^17^ However, inhalants can also have a wide range of peripheral effects throughout the body, including on the renal, cardiovascular, and pulmonary systems.^18^ Indeed, one of the most profound, but underexplored, consequences of inhalant abuse is its effect on energy balance. In humans, adolescent inhalant abuse is associated with weight loss,^19^, ^20^ and emaciation^21^; findings which are supported by meta‐analysis.^22^ This meta‐analysis found a significant dose‐response relationship to toluene‐induced weight impairments, with the lowest concentration band (0‐500 ppm) having no significant impact on body weight, suggesting that occupational level exposure does not impact body weight in the same way as inhalant abuse.^22^ Furthermore, one cohort study found that the weight suppressing effects of inhalant abuse was not mediated by other substance use, suggesting that inhalants are a strong mediator of this effect even with other drug‐drug interactions.^19^ Indeed, this effect on body weight is listed as a warning sign of inhalant abuse.^23^ Inhalant abuse in both males and females has been associated with disordered eating, including fasting, purging, and other behaviors associated with attempts to lose weight,^24^ suggesting that inhalants may be used as a means of suppressing appetite and thus body weight.^24^, ^25^ These findings are consistent with preclinical studies using rats, with adolescent and adult toluene exposure associated with reduced food intake, decreased weight gain, and reduced fat deposition.^26^, ^27^, ^28^, ^29^, ^30^

In addition to effects on food intake and body weight, exposure to toluene is associated with impaired linear growth.^22^ A clinical study of Indigenous males found that those with a history of chronic inhalant abuse in adolescence were, on average, 7 cm shorter than noninhalant users from the same communities^19^; this height impairment persisted into sustained abstinence.^31^ In weanling and adolescent rats, chronic toluene exposure at concentrations analogous to inhalant abuse impairs growth, with a significant reduction in rump width and body length.^26^, ^32^

The impact of inhalants on food intake, body weight, and growth is of importance given the association between inhalant abuse and adolescence. During adolescence, critical maturational processes occur, including the adolescent growth spurt,^33^ where skeletal structures grow and mature.^34^ Indeed, in humans, over half of the peak bone mass is attained during the adolescent growth spurt.^34^, ^35^ However, the adolescent growth spurt can be disrupted by factors such as malnutrition^36^ or exposure to estrogenic chemicals.^37^ Growth impairments can potentially recover if the harmful factor is removed, but if exposure occurs during adolescence usually only partial recovery is possible.^38^ Thus, any disruption to growth during adolescence has the potential to cause long‐term effects on growth patterns.^39^ Given that the highest rates of inhalant abuse are occurring during this critical period, studying the energy balance impacts of inhalant abuse specifically in adolescents, including the long‐term effects even if exposure ceases, is essential. This review will summarize the current understanding of the effects of inhalants on growth and energy balance.

## 4 AN OVERVIEW OF ENERGY BALANCE

**Figure 2:**
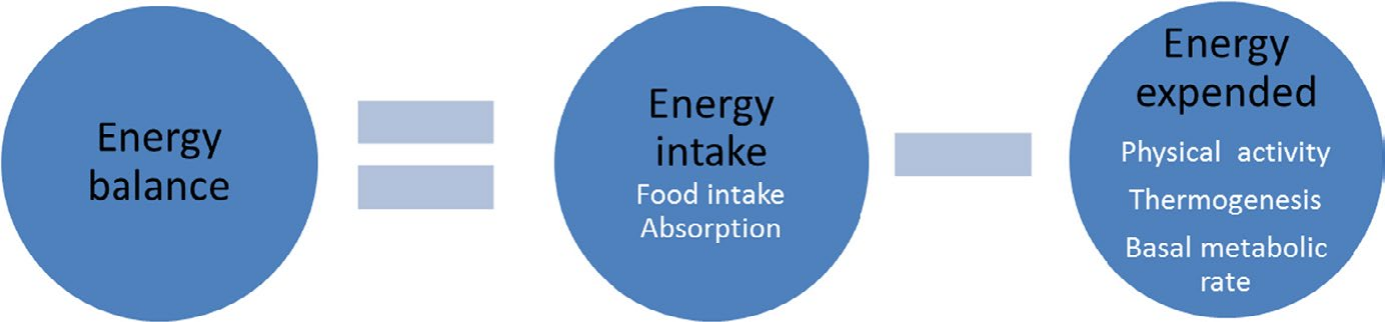
The energy balance equation. Figure by R Crossin, first cited in^108^

Food intake, body weight, and growth are intrinsically linked and can be represented via the energy balance equation (Figure 2). Energy intake is primarily driven by food intake and moderated by absorption and excretion. Energy expenditure includes basal metabolic rate, physical activity, and thermogenesis (subdivided into cold‐induced thermogenesis and the thermic effect of food). Body weight will remain stable when there is a balance between energy intake, storage, and expenditure; a process known as energy homeostasis.^40^ The maintenance of body weight is buffered, that is, a decrease in energy intake results in decreased expenditure and vice versa, therefore, any change in body weight occurs by overwhelming the buffering potential of homeostatic systems.^41^

Negative energy balance occurs when energy intake is less than the energy expenditure, and this shortfall is made up by a release of energy from body stores (eg, adipose tissue), leading to reduced body weight. If the difference between energy intake and energy expenditure is positive, then energy is stored and body weight increases (a positive energy balance). Physiological processes strongly defend reductions in body weight arising from negative energy balance, ultimately leading to weight regain.^42^ In contrast, positive energy balance leads to weight gain and does not initiate the same physiological defense, leading to obesity. This new increased body weight is subsequently defended.^43^ Energy balance becomes particularly important in periods of rapid growth, such as the adolescent growth spurt, with energy intake being one of the major determinants of growth.^44^ However, the effect of energy intake on growth is predominantly indirect, with growth impairments due to undernutrition primarily attributable to endocrine changes such as increased circulating cortisol.^45^, ^46^

Energy homeostasis, and thus body weight, is both centrally and peripherally regulated through the processes of metabolism,^40^, ^47^ as summarized in Table 1. When metabolic processes are functioning correctly, feedback mechanisms correct disruptions to energy homeostasis, with both the hypothalamus and brainstem sensing both short‐ and long‐term changes in energy balance. At first, the response to a negative energy balance is the promotion of appetite/feeding behavior, the utilization of glycogen stores, and when these are exhausted, the utilization of adipose tissue stores, and finally the breakdown of lean muscle mass. However, as adolescent inhalant abuse is associated with both decreased food intake, impaired weight gain, and fasting hypoglycemia,^26^, ^27^ this suggests that energy homeostasis is not being achieved and the feedback mechanisms, which should correct a negative energy balance, are dysfunctional.

**Table 1:** Central and peripheral regulation of energy balance

Key metabolic organ	Compound	Function
Pancreas	Insulin	Decreases glucose levels in the blood by glycogenesis and promotes lipogenesis (the storage of energy as adipose tissue)
Pancreas	Amylin	Slows gastric emptying and reduces appetite
Pancreas	Glucagon	Promotes gluconeogenesis and the release of glycogen from stores if circulating glucose levels become too low
Liver	Insulin	Responds to insulin secreted by the pancreas by taking up glucose from the blood
Kidney	Insulin	Clears and degrades circulating insulin
Gut	Ghrelin	Increases hunger and initiates feeding
Gut	Mechanical processes	Stretch receptors signal satiety to the brain via the vagus nerve
Gut	Peptide YY (PYY)	Slows gastric emptying and reduces appetite
Adipose tissue	Leptin	Opposes the actions of ghrelin, by sending an adiposity signal and inhibiting hunger and also inhibits lipogenesis
Brain	Receptors for gut hormones	Signals converge primarily on the brain stem and hypothalamus, which are the primary brain regions involved in maintaining energy homeostasis
Brain	Neuropeptide Y (NPY)	Increases food intake
Adrenal gland	Adrenalin and cortisol	Responds to hypoglycemia by inhibiting insulin release, and activates glycogenolysis and gluconeogenesis, via the HPA axis
Bone	Osteocalcin	Increases insulin production and sensitivity, and enhances glucose utilization

## 5 EFFECTS OF INHALANTS ON ENERGY BALANCE

Toluene has a complex pathway through the body. Once inhaled, toluene crosses rapidly from the lungs into the bloodstream via diffusion through the alveoli, before being metabolized by the liver and excreted by the kidneys.^48^ The primary metabolic pathway of toluene is the formation of benzyl alcohol, with further oxidation to benzaldehyde and benzoic acid, and then via conjugation to hippuric acid. Approximately 80% of absorbed toluene is excreted as hippuric acid in urine, with less than 1% excreted as o‐ and p‐cresol, and up to 14% excreted by exhalation.^49^, ^50^, ^51^ With repeated exposure, toluene can accumulate in organs, primarily the brain, adipose tissue, and adrenal glands,^48^, ^52^ which are key regions for metabolic processes, compared to limited accumulation in liver, kidney, and muscle tissue.^48^ Toluene can also cross^53^and disrupt^54^ the blood‐brain barrier and has a comparatively high accumulation in the brainstem and hypothalamus,^55^ both areas playing a critical role in the control of feeding and metabolism.^47^ This raises the potential that inhalant‐induced metabolic dysfunction may have both centrally and peripherally mediated components.

**Figure 3:**
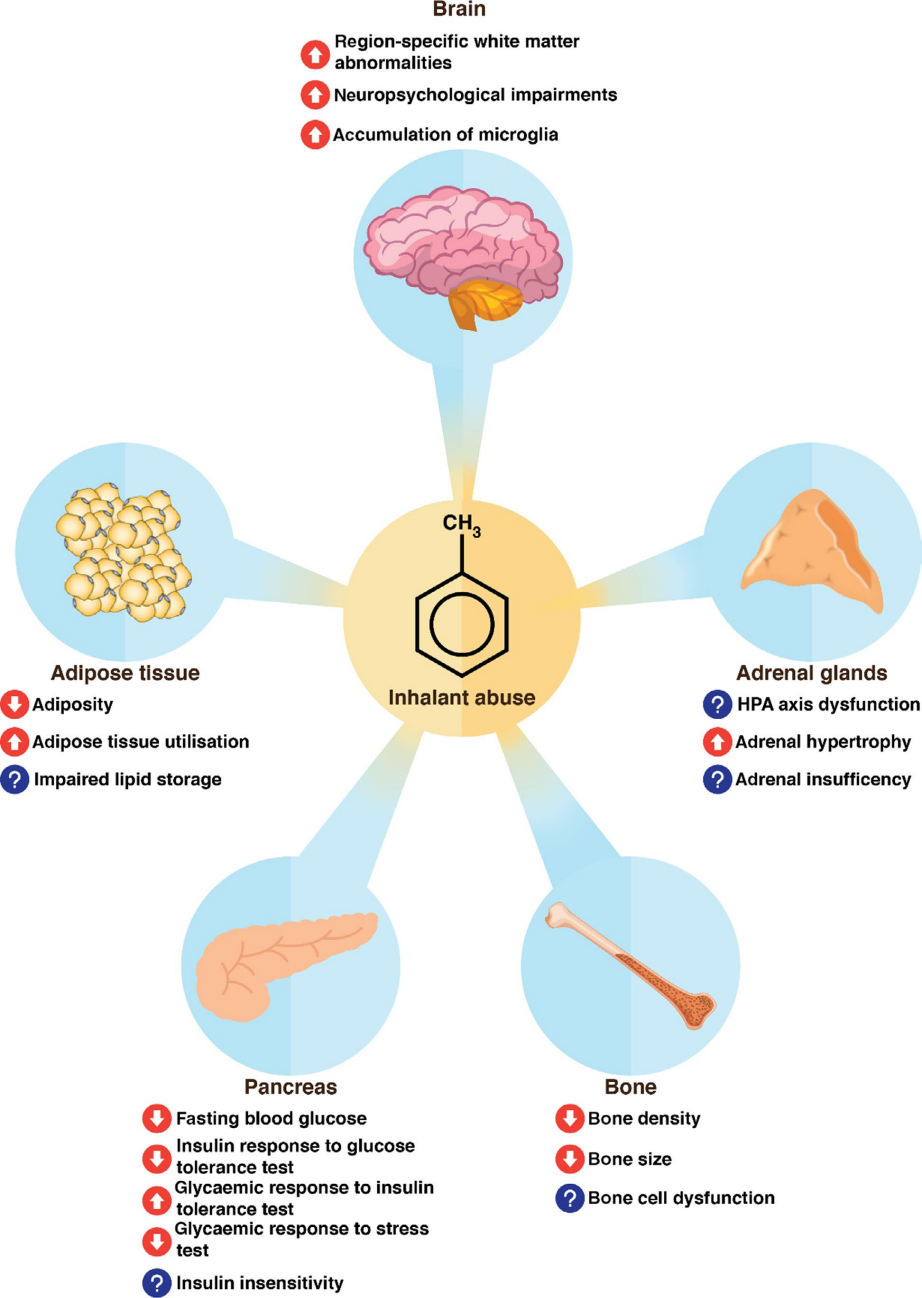
Key metabolic organs are affected by inhalant abuse, including the brain, pancreas, adipose tissue, bone, and adrenal glands

The symptoms of inhalant abuse (ie, reduced food intake, body weight suppression, and impaired growth) are suggestive of a negative energy balance. However, questions remain as to which components of the energy balance equation are altered, and how the central and peripheral regulation of energy balance may be affected. Theoretically, inhalant abuse could reduce energy intake through a direct effect on appetite, or by increasing energy expenditure via changes to physical activity, basal metabolic rate, or thermogenesis. Inhalant abuse may also disrupt the metabolic signaling that would promote appetite and weight gain in response to a negative energy balance. Alternatively, exposure to inhalants could also be directly affecting body composition through effects on the absorption or deposition of fat, or skeletal composition, which may explain growth impairments. Evidence suggests that inhalant abuse affects key metabolic organs (Figure 3), and each of these aspects of energy balance and energy balance regulation will be expanded upon in the following sections, as they are the key components to explore in order to understand the energy balance and growth effects of inhalant abuse.

## 6 EFFECTS OF INHALANTS ON ENERGY INTAKE

In humans, there is evidence that incidental inhalation of petrol fumes may have an acute anorectic effect, and in a food‐constrained environment, a desire to suppress feelings of hunger could be a driver for intentional petrol sniffing behavior.^56^ These data support previous anecdotal reports that petrol sniffing in Australian Indigenous communities is partly driven by a desire to suppress appetite.^25^ In adult rats, toluene‐induced anorexia has been attributed in part to the observed decrease in the hypothalamic expression of neuropeptide Y (NPY), a peptide which stimulates food intake.^30^ Furthermore, energy intake is moderated by absorption of nutrients occurring in the gut, and disruptions to absorption are known to be associated with other types of substance abuse^57^; however, we could find no studies that assessed nutrient absorption across the gut following toluene exposure or inhalant abuse. This is a potentially significant knowledge gap, given that inhalant users frequently report gastrointestinal disturbances, including nausea, vomiting, and diarrhea,^18^, ^58^ and that gut microbiomes have never been assessed in relation to inhalant abuse.

Evidence also suggests that inhalant abuse can impair metabolic signaling, because the normal responses to negative energy balance are impaired. Food intake is reduced in rodents following exposure to toluene at abuse concentrations, with animals exposed to toluene consuming fewer kilocalories than air‐exposed animals at the same body weight.^27^ This suggests an impairment in metabolic feedback processes, which should act to restore homeostasis in response to insufficient energy intake. In rats, adolescent toluene exposure alters the levels of gut hormones, including insulin, amylin, and peptide YY (PYY), and decreases hypothalamic leptin receptor mRNA expression.^27^ Importantly, decreased amylin and PYY levels should act to increase appetite and food intake, but this does not occur, supporting the idea of impaired metabolic signaling. This hypothesized impairment is further supported by evidence that food intake recovers in abstinence; however, body weight suppression continues,^26^ in contrast to the expected homeostatic recovery of body weight.^42^ These studies highlight that the observed growth changes, during both exposure and abstinence, are not directly correlated with food intake changes.

## 7 EFFECTS OF INHALANTS ON DIETARY PREFERENCES

**Figure 4:**
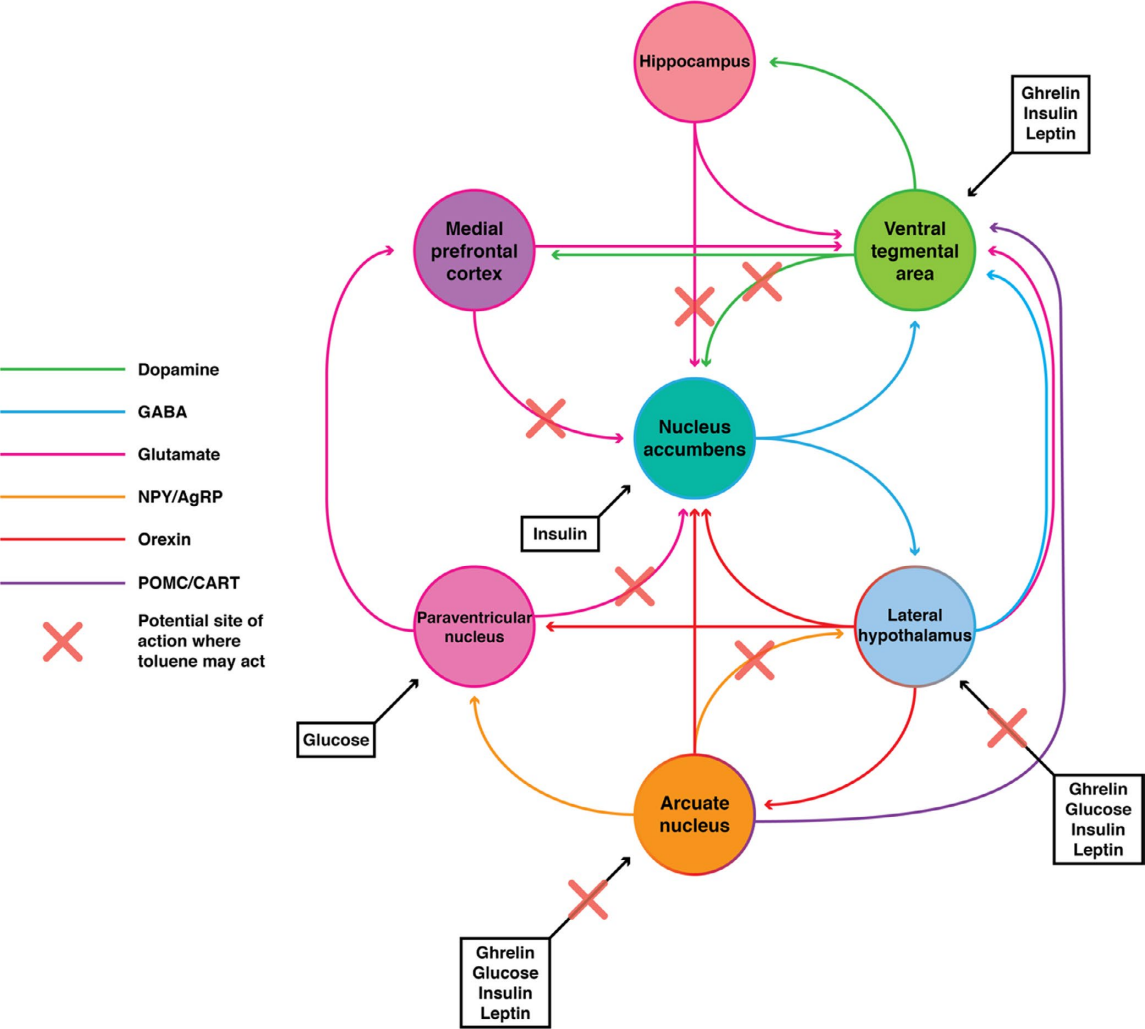
Inhalant abuse may alter both homeostatic and hedonic feeding pathways through multiple targets, including hypothalamic regulation of feeding and dopaminergic reward pathways. For simplicity, not all pathways, regions, or cell populations have been shown

In addition to changes in total energy intake, toluene has also been associated with alterations to dietary preferences and composition. Altered dietary intake alongside weight disruption has been reported in industrial workers chronically exposed to low toluene levels,^59^ though this has not been explored in a clinical cohort of people who misuse inhalants. In these individuals, exposure to toluene results in an increase in the consumption of total calories and carbohydrates, even though body mass index remains the same. As toluene is also known to act on dopaminergic pathways,^60^ it is possible that toluene may impact upon dopaminergic control of appetite and energy regulation (Figure 4). This may have long‐term ramifications, as dopaminergic pathways have a well‐established role in altering dietary preference^61^ including the potential to reinforce a high fat and high carbohydrate diet.^62^ Rats chronically exposed to high levels of toluene during adolescence exhibit a stronger preference for saccharin and for higher caloric chow compared to controls,^63^ potentially through long‐term consequences of toluene's effects on the mesocorticolimbic pathway (Figure 4). This may occur due to changes in hedonistic feeding pathways through alterations in reward signaling, or through the previously discussed homeostatic mechanisms to compensate for the body weight suppression that occurs during exposure. Altered glutamatergic signaling in the nucleus accumbens and dorsal striatum, through decreased NMDA receptor binding, was seen in a dose‐dependent manner in rats chronically exposed to high levels of toluene during adolescence.^27^ Toluene has also been shown to increase the level of dopamine in the nucleus accumbens in rats given a single dose,^64^ and in the ventral tegmental area in vivo through direct actions on dopaminergic neurons in this area.^65^

Future preclinical experiments using operant conditioning are required, both during active toluene exposure and abstinence, to systematically explore the effects of inhalants on dietary preferences and further characterize the role inhalants play in both hedonistic and homeostatic feeding, which could then be translated into clinical studies using nutritional assessment.

## 8 EFFECTS OF INHALANTS ON GLYCEMIC CONTROL

High fat and high carbohydrate diets have been associated with changes in glycemic control, and increase the risk for development of type 2 diabetes.^66^ This generally stems from ineffective insulin release and the development of insulin insensitivity, resulting in high, uncontrolled blood glucose levels that can have detrimental effects on the CNS, vision, and kidney function. Ultimately, if exposure to toluene alters glycemic control in association with a change in dietary preference, adolescent abusers could be predisposed to an increased risk of nutrition‐related disorders later in life. The potential that toluene‐induced metabolic dysfunction could increase the risk of diabetes is supported by studies of industrial workers exposed to low levels of toluene. These individuals were found to have higher than normal insulin^67^ and fasting blood glucose levels.^67^, ^68^ This suggests that chronic toluene exposure may play a role in the development of insulin resistance and thus, altered glycemic control^68^; the first step toward a diabetic phenotype, though the applicability in a clinical inhalant abuse context remains unknown.

In the clinical setting, a glucose tolerance test (GTT) is used to monitor how well glucose is metabolized and the capacity of the body to secrete adequate insulin. Typically, uncontrolled type 2 diabetic people have higher circulating glucose levels and a slower insulin response following a GTT. Adolescent rats chronically exposed to high doses of toluene demonstrated abnormal responses to GTT, including decreased insulin release.^27^ Abnormal GTTs continued to be observed in these rats during abstinence following a period of access to a high fat and high carbohydrate diet, with significantly increased glucose levels compared to control rats on the same diet.^27^ In contrast, however, adolescent rats chronically exposed to high concentrations of toluene exhibited low fasting blood glucose levels at the end of the exposure period, which could not be attributed solely to low food intake, as blood glucose levels were lower than pair‐fed controls.^26^ Additionally, these rats exhibited an increased glycemic response to an insulin tolerance test in comparison to controls, but an impaired glycemic response to a stress response test, with blood glucose disproportionately low compared to corticosterone response (which should promote an increase in blood glucose levels).^26^

The mechanisms by which toluene impairs glycemic control are unknown. It is possible that toluene is directly disrupting the activity of glucose‐sensing neuronal populations in the hypothalamus.^69^ By interfering with the activity of these neurons, toluene‐induced alterations to both feeding behavior and circulating hormone levels may occur. Hypothalamic damage has been reported in at least one patient chronically exposed to toluene during adolescence,^70^ suggesting that toluene may exert effects directly within the CNS. This may cause downstream effects to peripheral systems involved in glycemic regulation impairing the ability to sense and respond to changing glucose levels. It is also possible that these results reflect differing effects over the time course of inhalant abuse; with low fasting blood glucose as a more immediate effect, but decreased insulin sensitivity developing over time, potentially in response to increased dietary preferences for carbohydrates.

## 9 EFFECTS OF INHALANTS ON ENERGY EXPENDITURE

Energy expenditure in a clinical study of inhalant abuse has never been explicitly tested, but preclinical studies suggest that exposure to toluene affects components of energy expenditure. Toluene exposure in adolescents acutely increases locomotor activity,^71^ although this effect is transient and the response to toluene is biphasic with locomotor activity reduced at higher concentrations.^72^ In rats, adolescent exposure to toluene increased basal metabolism, particularly during the dark cycle.^26^ In contrast, earlier studies had showed that prenatal exposure to toluene resulted in reductions in metabolic rate and energy expenditure in the offspring, unfortunately this was not also assessed in the toluene‐exposed individuals.^29^ These studies also did not assess thermogenesis, in addition to physical activity or metabolic rate, but one study utilizing mice found that toluene acutely reduced core temperature in a dose‐dependent manner.^73^ Conversely, many inhalant users report a persistent low‐grade fever,^74^ although a mechanism for this remains unknown.

## 10 EFFECTS OF INHALANTS ON ADIPOSE TISSUE

Adipose tissue is the major site of long‐term energy storage in the body, and changes to adiposity will be reflected in changes to body weight. Therefore, adipose tissue is a key organ to consider in relation to adolescent inhalant abuse. Male adolescent rats exposed to toluene had significantly reduced adiposity but no difference in lean mass or total water^27^ and a propensity for increased adipose tissue utilization over carbohydrate utilization.^26^ Interestingly, in offspring that had prenatal exposure to toluene, body weight was also reduced, but toluene significantly increased adiposity in a dose‐dependent way in male, but not female, offspring.^29^ The increased adiposity was attributed to a compensatory developmental programming effect, whereby low birth weight alters hormonal and metabolic programming to maximize postnatal survival, and subsequently results in increased adiposity.^75^ Furthermore, when there is a negative energy balance caused by insufficient food intake, adipose stores are utilized by the body to provide energy. Thus, the observed decrease in adiposity^27^ associated with exposure to toluene may not be unexpected. However, alternative hypotheses as to why toluene reduces adiposity are that it affects the absorption of ingested fats within the digestive tract, or the ability to depose lipids within adipose tissue, such as that observed in pancreatic disorders.^76^ For example, both leptin and insulin play a crucial role in regulating lipogenesis^77^ and, therefore, the observed disruptions to hormones involved in energy balance signaling, including insulin,^27^ may impair the deposition of lipids in adipose tissue. While the studies discussed above show that exposure to toluene has an effect on body composition, particularly adiposity,^27^ it has yet to be determined whether toluene has a direct effect on body composition, or if these changes are indirect effects arising as a result of decreased food intake leading to a negative energy balance.

## 11 EFFECTS OF INHALANTS ON BONE

The reduction in weight gain arising from toluene exposure may not only be due to reduced adiposity, but also due to skeletal growth impairments. As well as a reduction in overall growth, inhalant abuse is associated with reduced bone density. A clinical study of adolescent males with a history of chronic glue sniffing found that bone mineral density was significantly reduced in comparison to an age‐matched nonsniffing group.^78^ However, no other human studies on the effects of adolescent inhalant abuse on skeletal growth could be found. Male adolescent mice exposed to toluene have significantly reduced bone mineral density compared to controls,^79^ although subsequent studies using rats did not find a reduction in bone density or skeletal microstructure following toluene exposure, rather that the bones were simply smaller.^26^

It is possible that the metabolic perturbations observed in inhalant abuse could be involved in the reported skeletal growth impairments. Leptin has a direct effect on bone cell function and acts to reduce bone fragility through anabolic effects on osteoblasts and the reduction of osteoclast formation.^80^ Amylin increases bone mass by inhibiting bone resorption.^81^ Insulin has an indirect effect on bone function, and increased circulating insulin is associated with increased bone density.^82^ All of these hormones are affected following exposure to toluene.^27^, ^67^ A further consideration is that bone itself can also exert reciprocal effects upon metabolism via osteocalcin, which increases insulin production and sensitivity, and enhances glucose utilization.^83^, ^84^ Osteocalcin can exert effects on both pancreatic β cells and adipocytes, making it a key component of metabolic regulation.^85^ In combination, these developments led to the conclusion that there is reciprocal regulation between energy metabolism and bone metabolism.^86^ Furthermore, this is mediated via the hypothalamus,^87^ which is known to be affected by exposure to toluene.^55^

## 12 EFFECTS OF INHALANTS ON THE HYPOTHALAMIC‐PITUITARY‐ADRENAL (HPA) AXIS AND ADRENAL GLAND

There is evidence to suggest that inhalant abuse may affect the HPA axis, though the nature of this relationship is equivocal. Some studies have linked inhalant abuse with an upregulation in HPA axis activity and associated behaviors,^88^, ^89^, ^90^ whereas others have found a blunted HPA response following inhalant abuse.^26^, ^91^, ^92^ It is also possible that there is a direct effect of toluene on the adrenal gland, which is vulnerable to toxicological injury due to high vascularity and fat content,^93^ particularly by aromatic hydrocarbons such as toluene.^94^ Multiple preclinical studies have found that adrenal hypertrophy is evident following inhalant abuse,^26^, ^27^, ^95^, ^96^ although only one clinical case study has associated inhalant abuse with an adrenal effect.^97^

Adrenal hypertrophy can be an outcome of chronic stress or adrenal insufficiency.^98^ Adrenal insufficiency is a failure to produce the stress hormone cortisol by the adrenal gland, either due to an issue in the adrenal gland (primary adrenal insufficiency) or impairment upstream in the HPA axis (secondary or tertiary adrenal insufficiency).^99^ Chronic stress is characterized by adrenal hypertrophy with elevated levels of cortisol, whereas adrenal insufficiency is characterized by adrenal hypertrophy with low or unchanged levels of cortisol, which is then further located within the HPA axis by the relative levels of adrenocorticotropic hormone (ACTH) and corticotropic‐releasing hormone (CRH).^98^, ^99^

When the adrenal gland has been directly investigated in relation to inhalant abuse, studies have found adrenal hypertrophy, particularly in the zona fasiculata where cortisol (corticosterone in rats) is produced, and low/unchanged levels of basal corticosterone with high levels of basal ACTH.^26^, ^95^, ^96^ Although these findings are diagnostically consistent with primary adrenal insufficiency,^99^ in two earlier studies the authors attributed their findings to chronic stress,^95^, ^96^ with a later study hypothesizing that adrenal insufficiency was present.^26^ Adrenal insufficiency is a chronic disorder with symptoms of decreased food intake, impaired body weight and growth, nondiabetic fasting hypoglycemia, persistent low‐grade fever, fatigue, and gastrointestinal disturbances.^99^ These symptoms are consistent with what has been observed following inhalant abuse,^20^, ^24^, ^26^, ^27^, ^58^, ^74^, ^100^ although they also fit a range of other disorders. Nevertheless, there is potential that HPA axis dysfunction, particularly adrenal insufficiency, may underlie the growth and energy balance consequences of inhalant abuse.

## 13 EFFECTS IN ABSTINENCE AND THE POTENTIAL FOR ADULT‐ONSET DISORDERS

Some of the energy balance effects arising from adolescent inhalant abuse, including reduced body weight and linear growth, persist into abstinence.^26^, ^27^, ^31^ Severe and chronic weight suppression is associated with cognitive impairments^101^ and health effects, including kidney and bone damage.^102^ Additionally, growth impairments are associated with adverse psychological consequences, including social anxiety and poor self‐image.^103^, ^104^ Conversely, weight regain after a period of weight suppression is associated with increased central adiposity and insulin resistance, both of which are risk factors for the development of metabolic syndrome, obesity, and type 2 diabetes.^42^, ^105^ Together this suggests that chronic toluene abuse could increase the risk of developing a diabetic phenotype, which is supported by human and rodent data linking impaired glucose tolerance to an increased risk of developing diabetes later in life. It is important to note, however, that it is currently equivocal whether weight suppression arising from adolescent inhalant abuse ultimately resolves, and this may be confounded in a clinical setting by other drug use during abstinence from inhalants.^31^ Nevertheless, adolescent inhalant abuse has the potential to initiate a sequence of long‐term metabolic alterations and thus lead to a chronic disease burden for individuals, even if exposure ceases. However, the nature of that disease burden remains unknown and knowledge on the health consequences of inhalant abuse during abstinence is scarce.

## 14 SUMMARY AND FUTURE WORK

The cognitive and neurological consequences of inhalant abuse have dominated inhalant abuse research. Long‐term cognitive impairment arising from intentional inhalation of inhalants containing toluene is well reported,^13^, ^14^, ^15^ although in extended abstinence, these effects generally improve and in some cases normalize completely.^28^, ^106^, ^107^ As discussed in this review, inhalant abuse is associated with decreased food intake, reduced body weight and linear growth, reduced adiposity, fasting hypoglycemia and altered glycemic regulation, altered levels of gut hormones, impaired HPA axis signaling as well as a preliminary link to impaired skeletal growth. These effects partially persist into sustained abstinence and cannot be solely attributed to reductions in food intake. Collectively, these symptoms suggest widespread energy balance dysfunction, which can impact growth and energy balance regulation, and may lead to an increased risk of chronic metabolic disorders for example, obesity and type 2 diabetes in adulthood, even if abuse ceases. However, the current state of research in this field does not enable determination of whether toluene predominantly affects the central or peripheral components of energy balance, nor enables us to determine the relative contributions of the effect on each organ, to the overall observed energy balance phenotype. Furthermore, the biochemical mechanisms underpinning these alterations have not yet been explored in detail.

These potential energy balance and growth impacts are not well‐researched, and the lack of longitudinal cohorts of inhalant users means that the long‐term health consequences of inhalant‐induced energy balance dysfunction are unknown. Furthermore, while a dose‐response relationship between toluene exposure and impaired body weight has been established, this has yet to be extended to other relevant endpoints, including changes to linear growth, dietary preferences, and metabolic signaling—such work would be highly valuable in distinguishing the impacts of occupational level exposure to toluene from inhalant abuse. Ultimately, we hope to stimulate additional research in this field, to further elucidate the long‐term health risks associated with adolescent inhalant abuse and assist clinicians to treat individuals with a history of inhalant abuse, subsequently improving their quality of life.

## ACKNOWLEDGEMENTS

This study was funded by the NHMRC, of which AJL is a Principal Research Fellow (1116930) and ZBA is a Senior Research Fellow (APP 1154974); RC was funded by an RTP Scholarship from the Australian Government. RC has received funding as an untied educational grant from Seqirus, unrelated to this study. We also acknowledge the Victorian Government's state infrastructure program.
